# In Vivo Electrophysiology of Peptidergic Neurons in Deep Layers of the Lumbar Spinal Cord after Optogenetic Stimulation of Hypothalamic Paraventricular Oxytocin Neurons in Rats

**DOI:** 10.3390/ijms22073400

**Published:** 2021-03-26

**Authors:** Daisuke Uta, Takumi Oti, Tatsuya Sakamoto, Hirotaka Sakamoto

**Affiliations:** 1 Department of Applied Pharmacology, Faculty of Pharmaceutical Sciences, University of Toyama, Toyama 930-0194, Japan; 2 Department of Biological Sciences, Faculty of Science, Kanagawa University, Hiratsuka, Kanagawa 259-1293, Japan; oti-bio@kanagawa-u.ac.jp; 3 Ushimado Marine Institute (UMI), Graduate School of Natural Science and Technology, Okayama University, Ushimado, Setouchi, Okayama 701-4303, Japan; ryu@okayama-u.ac.jp

**Keywords:** in vivo extracellular recording, in vivo whole-cell patch-clamp recording, optogenetics, spinal cord, lamina X, spinal ejaculation generator, gastrin-releasing peptide neurons, oxytocin

## Abstract

The spinal ejaculation generator (SEG) is located in the central gray (lamina X) of the rat lumbar spinal cord and plays a pivotal role in the ejaculatory reflex. We recently reported that SEG neurons express the oxytocin receptor and are activated by oxytocin projections from the paraventricular nucleus of hypothalamus (PVH). However, it is unknown whether the SEG responds to oxytocin in vivo. In this study, we analyzed the characteristics of the brain–spinal cord neural circuit that controls male sexual function using a newly developed in vivo electrophysiological technique. Optogenetic stimulation of the PVH of rats expressing channel rhodopsin under the oxytocin receptor promoter increased the spontaneous firing of most lamina X SEG neurons. This is the first demonstration of the in vivo electrical response from the deeper (lamina X) neurons in the spinal cord. Furthermore, we succeeded in the in vivo whole-cell recordings of lamina X neurons. In vivo whole-cell recordings may reveal the features of lamina X SEG neurons, including differences in neurotransmitters and response to stimulation. Taken together, these results suggest that in vivo electrophysiological stimulation can elucidate the neurophysiological response of a variety of spinal neurons during male sexual behavior.

## 1. Introduction

In the rodent spinal cord, there are several spinal centers that regulate the male sexual function. Truitt and Coolen [1,2] reported that disruption of galanin neurons at the L3–L4 level of the lumbar spinal cord did not change erectile capacity but eliminated only ejaculation, demonstrating that spinal galanin neurons are spinal ejaculation generator (SEG). The SEG neurons are located in lamina X and the medial part of lamina VII of lumbar spinal cord (L3–L4 level), expressing the neuropeptides, cholecystokinin [3,4], enkephalin [5], and gastrin-releasing peptide (GRP) [6] in addition to galanin. GRP neurons project their axons to the sacral autonomic nucleus and to the somatic spinal nucleus in the lower lumbar and the upper sacral spinal cord (L5–L6 and S1 level), which innervates bulbocavernosus and ischiocavernosus striated muscles attached to the base of the penis [7,8]. We have recently reported that hypothalamic oxytocin neurons project to the upper lumbar spinal cord (L3–L4 level) and are male-biased [9]. These axonal projections activate the SEG/GRP neurons, which express the oxytocin receptors (OXTR), and then influence penile reflexes, such as erection and ejaculation [9].

Electrophysiological studies of lamina X neurons have previously been carried out only in the spinal cord slices or in ex-vivo preparations [10,11]. Studies using slice preparations allow for the correlation of cellular morphology and neuropeptide distribution patterns with the electrophysiological/pharmacological response profiles of lamina X neurons. Further, ex-vivo preparations allow for the preservation of longitudinal architecture (e.g., intersegmental connections from cervical to sacral segments) and attached dorsal and ventral roots, which are essential for investigating the neural connections and vertical circuitry architecture of the spinal cord [12]. However, these preparations are unable to reveal neither in vivo functional characterization nor synaptic transmission of lamina X neurons. Specifically, ex-vivo preparations are methodologically limited to examine in a single slice along neuronal pathways like the brain–spinal cord neural circuit: hypothalamic oxytocin–SEG/GRP neural circuits. The purpose of this study is to develop a novel in vivo electrophysiological technique. First, we performed the in vivo extracellular recording from lamina X (SEG/GRP) neurons. Then, we performed in vivo whole-cell patch-clamp recordings from lamina X neurons to reveal the cellular characteristic of SEG/GRP neurons controlling male sexual function in response to oxytocinergic innervation from the paraventricular nucleus of the hypothalamus in rats.

## 2. Results

### 2.1. Newly Developed In Vivo Extracellular Recordings from Lamina X Neurons

**Figure 1:**
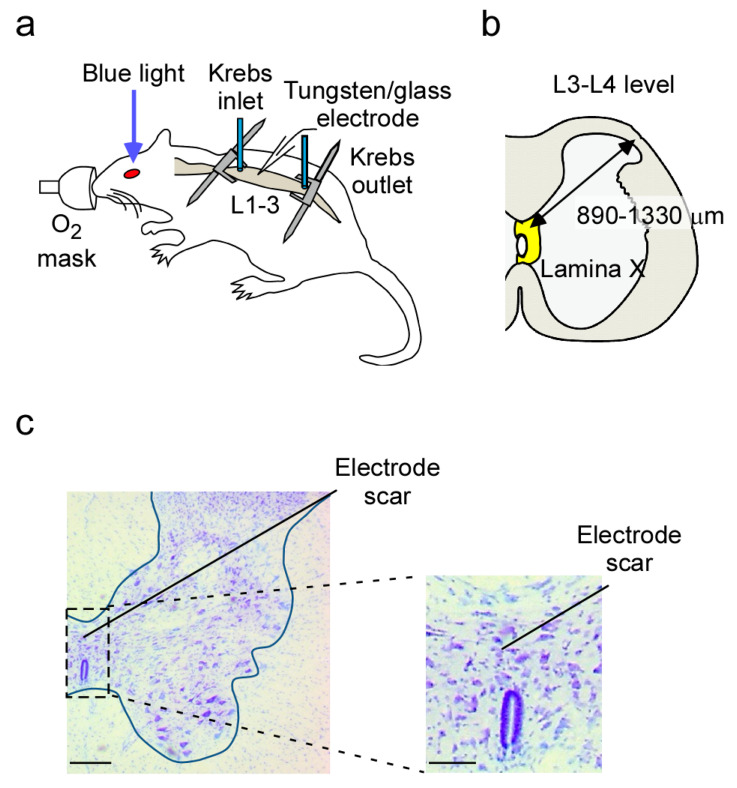
In vivo electrophysiological recording from lamina X neurons in the rat spinal cord. (**a**) Schematic diagram of the electrophysiological recording and perfusion system. The lumber spinal cord was exposed by laminectomy and the surface of the cord was perfused continuously with warmed pre-oxygenated Krebs solution. Known concentrations of drugs were applied from the same line. The body temperature was monitored and kept at the normal range. (**b**) Schematic drawing of the transverse slice of lumbar cord and of the angle of a recording electrode (two-way arrow). Recordings were made from cells at a depth of 890–1330 μm (shown by two-way arrow) from the surface of spinal cord. (**c**) A representative image of the Nissl-stained spinal cord section after insertion of a tungsten electrode. The blocked area is enlarged. Scale bars, 100 µm in the low magnification, 50 µm in the high magnification.

**Figure 2:**
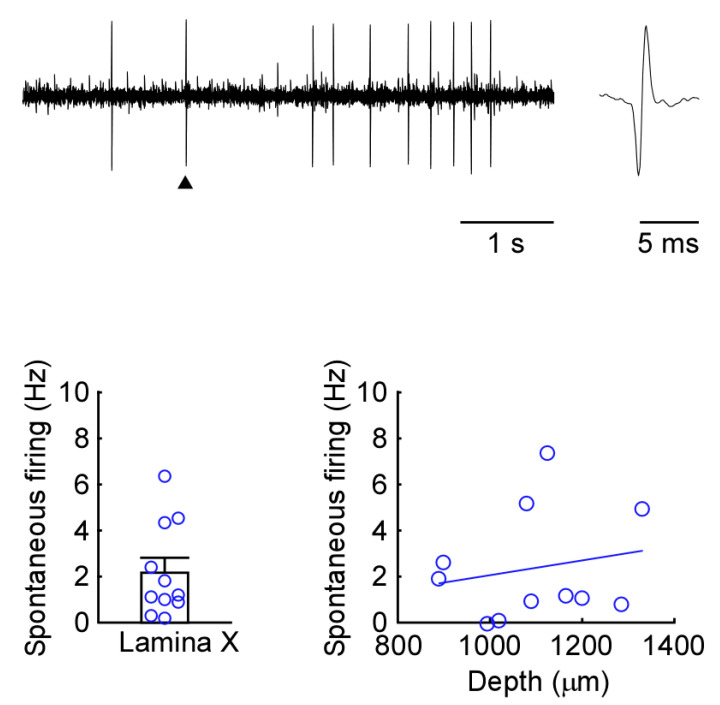
In vivo extracellular recording from lamina X neurons in the rat spinal cord. Representative trace of spontaneous firing recorded from a lamina X neuron (upper panel). The spike shape of an action potential, indicated by an arrowhead, is enlarged on the right side of the trace. Summary showing the frequency of spontaneous firing (lower panel; left) and correlations between the frequency and recording depth in the spontaneous firing (lower panel; right).

Anesthetized rats were maintained in a stable condition by supplying oxygen through a mask. If a withdrawal reflex appeared, then a supplemental dose of urethane was given during surgery and the data collection. A thoracolumbar laminectomy was performed exposing the level from Th11 to L4, and the animal was then placed in a stereotaxic apparatus (Figure 1a,b). An animal preparation could be maintained in a stable condition for at least 6 h. We first performed the conventional extracellular technique to record spontaneous firing from neurons in the lamina X (Figure 1c). These neurons showed a stable spontaneous discharge with an average frequency of 2.2 ± 0.6 Hz (*n* = 11 neurons from 5 rats, Figure 2). Recordings were obtained from lamina X neurons at a depth of average of 1098 ± 43 μm from the surface (*n* = 11 neurons from 5 rats, Figure 2).

### 2.2. Newly Developed In Vivo Whole-Cell Patch-Clamp Recordings from Lamina X Neurons

**Figure 3:**
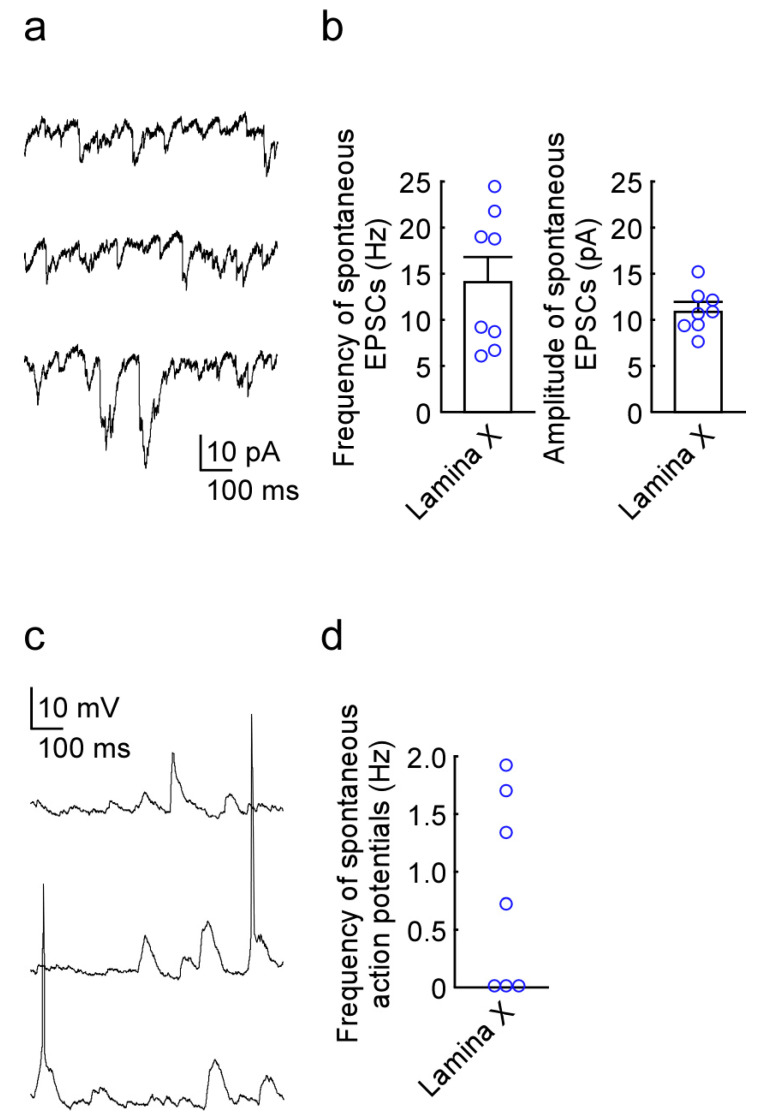
In vivo patch-clamp recording from lamina X neurons in the rat spinal cord. (**a**) Representative trace of spontaneous excitatory postsynaptic currents (sEPSCs) recorded from a lamina X neuron. (**b**) Summary showing the frequency (left) and the amplitude (right) of sEPSCs. (**c**) Under current clamp condition, representative trace of spontaneous excitatory postsynaptic potentials and action potentials recorded from a lamina X neuron. (**d**) Summary showing the frequency of spontaneous action potentials.

Stable whole-cell patch-clamp recordings were made from 8 lamina X neurons. The resting membrane potential of the lamina X neurons was −63.9 ± 1.3 mV (*n* = 8 neurons from 4 rats). Under voltage-clamp conditions at a holding potential of −70 mV, lamina X neurons exhibited spontaneous excitatory postsynaptic currents (sEPSCs) with an average amplitude of 11.0 ± 0.78 pA and a frequency of 14.2 ± 2.6 Hz (*n* = 8 neurons from 4 rats, Figure 3a,b). In the current clamp condition, a portion of lamina X neurons exhibited spontaneous action potential discharge (*n* = 7 neurons from 4 rats, Figure 3c,d).

### 2.3. In Vivo Extracellular Recordings after Optogenetic Stimulation of the Paraventricular Nucleus of the Hypothalamus (PVH)

**Figure 4:**
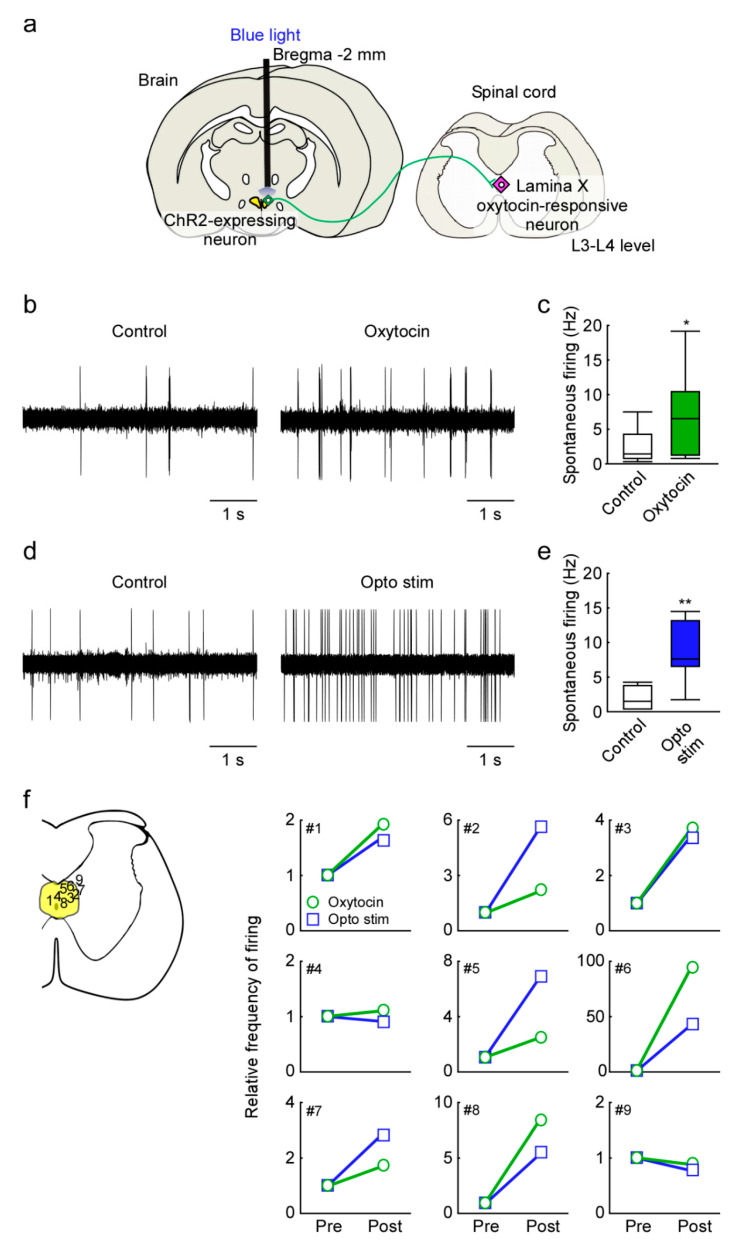
In vivo extracellular recordings from lamina X oxytocin-responsive neurons in the upper lumbar spinal cord (L3–L4 level) after superfusion of oxytocin or optogenetic stimulation of the paraventricular nucleus of the hypothalamus (PVH) of *Oxtr*-ChR2-EYFP transgenic rats. (**a**) Schematic drawing of the brain–spinal cord neural network controlling male sexual function and the location of optogenetic stimulation. The oxytocin neurons, expressing channel rhodopsin (ChR2; green) in the paraventricular nucleus of the hypothalamus project to the upper lumbar spinal cord (L3–L4 level) and act on the lamina X oxytocin-responsive neurons (magenta). (**b**) In vivo electrophysiology revealed that oxytocin-responsive neuronal firing was increased by oxytocin superfusion (1 μM) compared to control. (**c**) Quantification analysis showed that spontaneous firing was increased after oxytocin superfusion compared with control (the boxes show the 25th to 75th percentiles and the median, *n* = 9; paired *t*-test, * *p* < 0.05). (**d**) In vivo electrophysiology revealed that the frequency of oxytocin-responsive neuronal firing was significantly increased by the optogenetic stimulation of PVH when compared with control. (**e**) Quantification analysis showed that spontaneous firing was increased after optogenetic stimulation compared with control (the boxes show the 25th to 75th percentiles and the median, *n* = 7; paired *t*-test, ** *p* < 0.01). (**b**,**d**) were recorded from the same neuron. (**f**) Responsiveness of superfusion of oxytocin or optogenetic stimulation on spontaneous firing from lamina X neurons. (Left) Summary of recording depth on each neuron (plotted by numbers: 1–9). (Right) Summary showing effect of oxytocin or optogenetic stimulation on the relative frequency of firing in lamina X neurons.

We examined in vivo extracellular recordings from lamina X oxytocin-responsive neurons in the upper lumbar spinal cord (L3–L4 level) after superfusion of oxytocin or optogenetic stimulation of the PVH of Oxtr promoter-human heparin-binding epidermal growth factor-like growth factor (human diphtheria toxin receptor; Dxtr)-channel rhodopsin (ChR2)-EYFP BAC (Oxtr-ChR2-EYFP) transgenic rats (Figure 4a). It has been confirmed that OXT neurons expressing OXTR in the PVH are activated by optogenetic stimulation of PVH [9]. If the recorded lamina X neurons produced firing by bath applications of oxytocin, we regarded those as oxytocin-responsive neurons. Bath applications of oxytocin (1 μM) significantly increased the spontaneous firing of most lamina X neurons examined (from 2.43 ± 0.74 to 7.15 ± 1.98 Hz, *n* = 9 neurons from 5 rats, *p* < 0.05, Figure 4b,c,f). The frequency of lamina X neuronal firing was significantly increased by the optogenetic PVH stimulation (from 1.99 ± 0.61 to 8.49 ± 1.62 Hz, *n* = 7 neurons from 5 rats, *p* < 0.01, Figure 4d–f), demonstrating that in vivo activation of PVH oxytocin neurons facilitates activity in lamina X neurons of the lumbar cord via axonal oxytocin release within the spinal cord. Oxytocin-unresponsive neurons did not increase firing after optogenetic PVH stimulation (from 4.1 ± 2.7 to 3.6 ± 2.5 Hz, *n* = 2 neurons from 5 rats, Figure 4f).

## 3. Discussion

**Figure 5:**
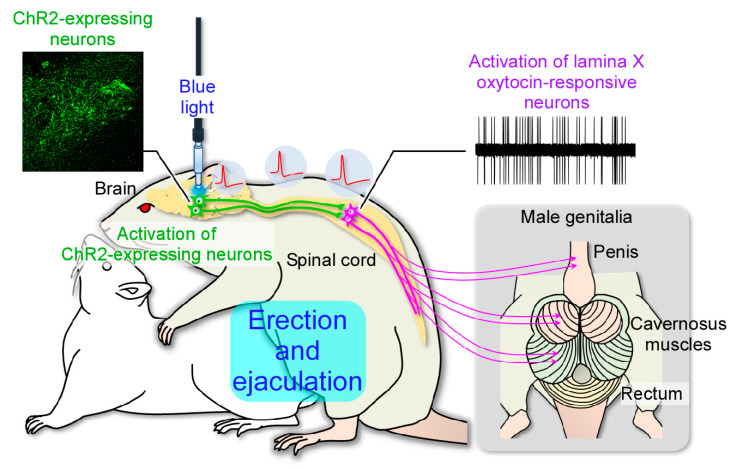
Schematic drawing summarizing the brain–spinal cord neural network that controls the male sexual function. A group of oxytocin neurons located in the posterior part of the paraventricular nucleus of the hypothalamus (PVH) project to the lower spinal cord and control penile erection and ejaculation in male rats. The lamina X oxytocin-responsive neurons containing gastrin-releasing peptide (GRP) project axons to spinal centers of the lumbosacral cord that mediate penile reflexes and trigger ejaculation.

In this study, we showed that activation of oxytocin neurons in the hypothalamus increases spontaneous firing of lamina X neurons in the lumbar spinal cord in vivo. In vivo electrophysiological analysis in the spinal cord has previously been performed on spinal dorsal horn neurons involved in the somatosensory such as pain and itch [13,14,15]. Electrophysiological analysis of neurons in the deeper regions (lamina VII and X) has only been performed by slice preparation or ex-vivo preparation [10,11,12]. Therefore, the synaptic inputs to lamina VII and X neurons and in vivo functional characterization remain unknown. Here, we have succeeded in establishing a novel technique for analyzing the in vivo electrical response of lamina X neurons in the spinal cord. Consequently, we have demonstrated the brain–spinal cord neural circuit that controls male sexual function in vivo for the first time (Figure 5).

Previous reports using in vivo extracellular recordings and in vivo whole-cell recordings show that synaptic response could be obtained from the superficial spinal dorsal horn neurons [13,16,17,18]. Recording from deeper regions of the spinal cord have only been demonstrated using slice preparation or ex-vivo preparation of the cord [12]. There are no studies that use in vivo recording techniques from deeper region of spinal cord (e.g., lamina X) because target neurons could not be identified in the deeper region of the spinal cord. In the present study, focusing on the expression of oxytocin receptors in GRP neurons, we identified GRP neurons using the response to oxytocin. Although lamina X neurons in the lumbosacral cord are thought to be key integrators for visceral somatosensory and nociceptive inputs and play an important role in male sexual function, such as erection and ejaculation, the functional properties of lamina X neurons and their connections remain poorly understood due to limited access. Therefore, it was difficult to reveal the visceral afferents regulating visceral pain and spinal neuronal circuits controlling male sexual function. Here, we obtained, for the first time, the spontaneous frequency of action potential of lamina X neurons in the lumbar cord using both extracellular recordings and whole-cell patch-clamp recordings in vivo. Furthermore, in vivo extracellular recording from the lamina X of the lumbar cord demonstrated the response of lamina X neurons to neurotransmitters (e.g., glutamate or GABA) in addition to the peptidergic signaling. To the best of our knowledge, this is also the first demonstration of in vivo recordings from deeper region of the spinal cord. Such in vivo recording techniques would be useful for studying in the visceral somatosensory system and intraspinal neuronal circuits controlling a variety of urogenital functions. However, it is still in the early stage of development. In the future, we need to proceed with more detailed analyses and analyze the neural responses from the lamina X neuron.

Projections of PVH oxytocin neurons to the lumbar cord have been well documented and are limited to projections from oxytocin neurons in the posterior part of the parvocellular PVH [19,20]. We have now demonstrated that optogenetic stimulation of Oxtr- and ChR2-expressing neurons in the PVH leads to activation of GRP neurons. Given that (1) oxytocin neurons in the PVH are sensitive to oxytocin [21] and that (2) the PVH also contains a similar number of oxytocin-insensitive vasopressin neurons [22] and parvocellular neurons projecting largely to the median eminence but not to the spinal cord [23], our data indicate that the majority of OXTR-expressing (i.e., ChR2+ and EYFP+) neurons in the PVH that project to the spinal cord are oxytocin neurons. Furthermore, in the lumbar cord, most oxytocin-immunoreactive axons are located in the medial region of lamina X where the GRP neurons are located. Approximately, 93% GRP neurons express EYFP and therefore also express oxytocin receptors [9]. Thus, there appears to be substantial evidence for an effect on GRP neurons of the PVH oxytocin neurons that project to the lumbar cord [19,20,24].

The mechanisms underlying the somatosensory system have been widely investigated with electrophysiological techniques. In contrast, the mechanisms underlying visceral pain remain unclear. It is known that visceral afferent sensory information is innervated to X layer neurons [25,26,27], but little electrophysiological analysis has been performed. It should be possible to clarify the mechanism of action of visceral pain using in vivo electrophysiological analysis. Furthermore, it is thought to be useful for elucidating the pain mechanism, due to cystitis, external genital pain, and paresthesias due to urogenital disorders, and for developing the new therapeutic agents. Moreover, systemic administration of the amphetamine analog p-chloroamphetamine, an indirect serotonergic agonist, has been reported to induce ejaculation both in awake [28,29] and anesthetized rats [30]. Penile erections in rats can also be evoked by the odorant (pheromone) stimulation from estrous females [31]. Using an in vivo extracellular recording from male rats induced ejaculation and/or noncontact erections, we may analyze whether ejaculation and/or erections activate lamina X (SEG/GRP) neurons.

## 4. Materials and Methods

All experiments were performed in accordance with the “Guiding Principles for Care and Use of Animals in the Field of Physiological Sciences” of the Physiological Society of Japan and were approved by the local Animal Experiment Committees of the University of Toyama and Okayama University. All efforts were made to minimize animal suffering and the number of animals used for the studies.

### 4.1. Animals

Adult male Oxtr promoter-Dxtr-ChR2-EYFP BAC (Oxtr-ChR2-EYFP) transgenic rats [9] (Wistar strain) bred in the animal facilities of Okayama University were used. Adult male wild-type (Wt) rats of the Wistar strain (Charles River Japan, Yokohama, Japan) were also used. All rats were maintained on a 12-h light/dark cycle and provide with unlimited access to water and rodent chow.

### 4.2. In Vivo Preparation

The adult male Wistar rats were anesthetized with urethane (1.2–1.5 g/kg, i.p.). Urethane produces a long-lasting steady level of anesthesia, which does not require administration of additional doses except in a few cases. A thoracolumbar laminectomy was performed exposing the levels from Th11 to L4, and the animal was then placed in a stereotaxic apparatus. After removing the dura and cutting arachnoid membrane to make a window large enough to let a tungsten microelectrode, the surface of spinal cord was irrigated with 95% O_2_–5% CO_2_-equilibrated Krebs solution (10–15 mL/min) containing the following (in mM): 117 NaCl, 3.6 KCl, 2.5 CaCl_2_, 1.2 MgCl_2_, 1.2 NaH_2_PO_4_, 11 glucose, and 25 NaHCO_3_, through glass pipettes at 37 °C ± 1 °C.

### 4.3. In Vivo Extracellular Recording from Lamina X Neurons

Standard extracellular single-unit recordings were performed as described previously [9,14]. Recordings were all obtained from lamina X neurons, dorsal to the central canal, at a depth of 890–1330 μm from the dorsal surface. These cells were within the lamina X as assessed by slices obtained from the same spinal level of age-matched rats (see Figure 1 and Figure 5). Unit signals were acquired with an amplifier (EX1; Dagan corporation, Minneapolis, MN, USA). The data were digitized with an analog to digital converter (Digidata 1400A, Molecular Devices, Union City, CA, USA), stored on a personal computer with a data acquisition program (Clampex version 10.2; Molecular Devices), and analyzed with a special software package (Clampfit version 10.2; Molecular Devices).

### 4.4. In Vivo Patch-Clamp Recording from Lamina X Neurons

Standard blind whole-cell voltage-clamp recordings were obtained as previously described [15]. The electrodes used in this study were pulled from thin-walled borosilicate glass capillaries (o.d. 1.5 mm) using a puller (p-97, Sutter Instrument, Novato, CA, USA) and were filled with a solution containing potassium gluconate solution (in mM): K-gluconate 135, KCl 5, CaCl_2_ 0.5, MgCl_2_ 2, EGTA 5, HEPES 5, and ATP-Mg 5 (pH 7.2). The tip resistance of the patch pipettes was 6–12 MΩ. As shown in Figure 1, the electrode was advanced at an angle of 30° into the lamina X through the window using a micromanipulator (Model MHW-4-1, Narishige, Tokyo, Japan), and a gigaohm seal was then formed with a cell at a regular depth of 890–1330 μm measured from the point of contact with the cell to the dorsal surface of the spinal cord. This distance was within the lamina X, which was identified using transverse slices obtained from the spinal cords of 7- to 10-week-old rats in the same level (Figure 1c). Series resistance was assessed according to the response to a 5-mV hyperpolarizing step. This value was monitored during the recording session, and data were rejected if values changed by >15%. Signals were acquired with a patch-clamp amplifier (Axopatch 200B, Molecular Devices). The data were digitized with an analog to digital converter (Digidata 1400A, Molecular Devices), stored on a personal computer using a data acquisition program (Clampex version 10.2, Molecular Devices), and analyzed using a software package (Clampfit version 10.2, Molecular Devices). Cell recordings were made in voltage-clamp mode at holding potentials of −70 mV to record EPSCs [15]. At first, the responsiveness of lamina X neurons to oxytocin application was tested. Control was measured for 30 s at 2 min before oxytocin application and/or blue light stimulation, and then, effects of oxytocin and/or blue light stimulation were evaluated for 30 s at 10 min thereafter. Frequency was calculated from measurements for 30 s in all experiments.

### 4.5. Oxytocin Superfusion

We first identified electrophysiologically oxytocin-responsive lamina X (GRP) neurons in the lumbar cord after the oxytocin superfusion. The identification was based on the fact that the lamina X neurons can respond to oxytocin. The surface of the spinal cord was irrigated with 95% O_2_/5% CO_2_-equilibrated Krebs solution (10–15 mL min^−1^) containing oxytocin (1 µM, 2 min; AnaSpec Inc., Fremont, CA, USA), through glass pipettes at 37 ± 1 °C.

### 4.6. Optogenetics

Optogenetic stimulation of PVH was an established method [9]. For in vivo electrophysiological analysis of lamina X neurons in the lumbar spinal cord (L3–L4 level) during either superfusion of oxytocin to the lumbar cord (*n* = 9 neurons from 5 rats) or blue light stimulation (*n* = 9 neurons from 5 rats) to the PVH oxytocin neurons to induce axonal oxytocin secretion in the lumbar spinal cord, Oxtr-ChR2-EYFP transgenic male rats were implanted with an optical fiber (NA 0.50; THORLABS, Newton, NJ, USA) targeting the posterior part of the left PVH (2 mm posterior to bregma, 1 mm left lateral to midline, 8 mm ventral to skull surface). The optical fiber was connected to an optical swivel (RJPSF-SMA, THORLABS) and then connected to a laser light source (470 nm; M470F3, THORLABS) that was controlled by either a schedule stimulator (Lucir Inc., Tsukuba, Japan) or without stimulation (control). In vivo extracellular recordings during superfusion of oxytocin (1 µM, 2 min; AnaSpec Inc., Fremont, CA, USA) or blue light stimulation (at least 10 mW, 15 ms light pulses, 20 Hz) were performed as described below.

### 4.7. Recording Position

To confirm the position of electrode insertion, we used the Nissl staining. A tungsten needle was inserted at a depth of about 1000 μm from the dorsal surface. After the recordings, rats were deeply anesthetized with intraperitoneal injections of sodium pentobarbital (50 mg kg^−1^ body weight) and perfused via the left ventricle with 100 mL of physiological saline followed by 200 mL of 4% formaldehyde in 0.1 M phosphate buffer (PB; pH 7.4). Spinal cords were immediately removed and post-fixed in the same fixative for 3 h at room temperature. After immersion in 25% sucrose in 0.1 M PB for 48 h at 4 °C for cryoprotection, lumbar cords were quickly frozen using powered dry ice and cut into 30-μm thick cross-sections (the lumbar cord: L3–L4 level) on a cryostat (CM3050 S, Leica, Nussloch, Germany). Nuclei in these sections were stained with Cresyl violet Nissl stain (Muto Pure Chemicals CO., LTD, Tokyo, Japan). Sections were imaged with an all-in-one fluorescence microscope and cellSens software (FSX100, Olympus, Tokyo, Japan).

### 4.8. Statistical Analysis

All data values were expressed as mean ± S.E.M. Statistical significance was determined as *p* < 0.05 using Student’s paired *t*-test when comparing two groups. In all cases, *n* refers to the number of neurons studied.

## 5. Conclusions

In this study, we demonstrated, by using in vivo electrophysiology recordings, the characteristics of the brain–spinal cord neural circuit controlling male sexual function. To our knowledge, this is the first demonstration of a technique for analyzing the in vivo electrical response from the deeper (lamina X) neurons in the spinal cord. Furthermore, we succeeded in the in vivo whole-cell recordings of lamina X neurons. In vivo whole-cell recordings during male sexual activity may reveal the features of lamina X (SEG/GRP) neurons such as the differences of neurotransmitters and those of response to stimulation. Taken together, these approaches enable us to understand the neurophysiological response of a variety of spinal neurons during male sexual behavior. Establishing the in vivo electrophysiological recording methods from the deeper region of the spinal cord, however, we might have many problems (e.g., cell type-specific identification and/or awake condition), and further studies are required to draw a firm conclusion.

## Data Availability

Not applicable.

## Abbreviations

ChR2	Channelrhodopsin-2
eYFP	enhanced yellow fluorescent protein
Dxtr	human diphtheria toxin receptor
Wt	wild-type
SEG	spinal ejaculation generator
GRP	gastrin-releasing peptide
OXTR	oxytocin receptor
PVH	paraventricular nucleus of the hypothalamus
sEPSCs	spontaneous excitatory postsynaptic currents
